# Intention to breastfeed and awareness of health recommendations: findings from first-time mothers in southwest Sydney, Australia

**DOI:** 10.1186/1746-4358-4-9

**Published:** 2009-10-16

**Authors:** Li Ming Wen, Louise A Baur, Chris Rissel, Garth Alperstein, Judy M Simpson

**Affiliations:** 1 School of Public Health, Sydney Medical School, University of Sydney, Australia; 2 Health Promotion Service, Sydney South West Area Health Service, New South Wales, Australia; 3 Discipline of Paediatrics & Child Health, University of Sydney, Australia; 4 School of Medicine Sydney, University of Notre Dame, Australia

## Abstract

**Background:**

In 2001, the World Health Organisation (WHO) recommended exclusive breastfeeding for the first six months of life. The objectives of this study are to assess awareness of the WHO recommendation among first-time mothers (women at 24 to 34 weeks of pregnancy) and to explore the relationship between this awareness and mothers' intention to exclusively breastfeed for six months.

**Methods:**

This study was part of the Healthy Beginnings Trial (HBT) conducted in southwest Sydney, Australia. We analysed cross-sectional baseline data of the trial conducted in 2008, including 409 first-time mothers at 24 to 34 weeks of pregnancy. The mothers' awareness of the recommended duration of exclusive breastfeeding and their intention to meet the recommendation were assessed through face-to-face interviews. Socio-demographic data were also collected. Factors associated with awareness of the recommendation, or the intention to meet the recommendation, were determined by logistic regression modeling. Log-binomial regression was used to calculate adjusted risk ratios (ARR).

**Results:**

Sixty-one per cent of mothers knew the WHO recommendation of exclusive breastfeeding for six months. Only 42% of all mothers intended to meet the recommendation (breastfeed exclusively for six months). Among the mothers who knew the recommendation, 61% intended to meet the recommendation, compared to only 11% among those mothers who were not aware of the recommendation.

The only factor associated with awareness of the recommendation was mother's level of education. Mothers who had a tertiary education were 1.5 times more likely to be aware of the recommendation than those who had school certificate or less (ARR adjusted for age 1.45, 95% CI 1.08, 1.94, p = 0.02). Mothers who were aware of the recommendation were 5.6 times more likely to intend to breastfeed exclusively to six months (ARR adjusted for employment status 5.61, 95% CI 3.53, 8.90, p < 0.001).

**Conclusion:**

Awareness of the recommendation to breastfeed exclusively for six months is independently associated with the intention to meet this recommendation. A substantial number of mothers were not aware of the recommendation, particularly among those with low levels of education, which is of concern in relation to promoting breastfeeding. Improving mothers' awareness of the recommendation could lead to increased maternal intention to exclusively breastfeed for six months. However, whether this intention could be transferred into practice remains to be tested.

**Trial Registration:**

HBT is registered with the Australian Clinical Trial Registry (ACTRNO12607000168459)

## Background

Breastfeeding has a wide range of health benefits for mothers and children and is a key protective factor against childhood overweight and obesity [1-4]. The current World Health Organization (WHO) recommendation for breastfeeding is that all infants should be exclusively breastfed for the first six months of life, and receive nutritionally adequate and safe complementary foods while breastfeeding continues for up to two years of age or beyond [2]. The WHO recommendations have been adopted and endorsed by many countries including Australia [3,5].

In Australia, a national survey found that in 2004-5, breastfeeding initiation was 88% [6], and similarly in the state of New South Wales (NSW), the percentage of infants "ever breastfed" was 90% in 2001 [7] and 87% in 2003-4 [8] respectively. However, this high initiation rate of breastfeeding does not lead to a high prevalence of sustained breastfeeding: only 16% of infants were exclusively breastfed to six months and 29% were breastfed to 12 months [8]. The Health Department of NSW recommends the promotion of breastfeeding for all mothers and infants to focus on extending the duration of breastfeeding to 12 months and exclusive breastfeeding to six months, in particular among those mothers who are most socio-economically disadvantaged, less than 25 years of age, or with less than a tertiary education [8].

Breastfeeding decisions and practices are influenced by multiple factors including knowledge, attitudes and beliefs, as well as socio-cultural and physiological factors [9-13]. However, results from research into determining these factors to date have been very variable due to a lack of objective, reliable, valid and sensitive measures [9]. In developed countries like Australia, mothers who are younger (under 25 years old), have less education, or are most socio-economically disadvantaged tend to have lower rates of full breastfeeding, rates of initiation and duration of breastfeeding [8,14].

There is increasing recognition of the need to promote exclusive breastfeeding since the WHO recommendation on exclusive breastfeeding for six months was made in 2001. However, the impact of such promotion on rates of exclusive breastfeeding is less clear [15]. This might be explained by a poor understanding of the breastfeeding recommendation, and of knowledge, attitudes and practice about breastfeeding in the community.

Research has repeatedly found that women's pre-birth breastfeeding intentions are a good predictor of the actual duration of breastfeeding [16,17]. In a study conducted in a group of Australian women, Rempel found that a strong desire to breastfeed was positively associated with breastfeeding at six months and having no intention to breastfeed was negatively associated with breastfeeding at six months [18].

To enhance breastfeeding promotion strategies in the context of relatively recently changed recommendations, it is important to have a good understanding of mothers' knowledge of the current recommendation on breastfeeding and their intention to meet the breastfeeding recommendation. In 2008, we commenced the Healthy Beginnings Trial (HBT) to test the effectiveness of an early childhood obesity intervention in the first two years of life [19]. The intervention uses a home-visiting strategy to promote healthy feeding of babies among first-time mothers. As part of this trial, we aimed to increase exclusive breastfeeding for the first six months among participating mothers.

This paper reports on those aspects of the data collected for the HBT that were collected at the baseline interview, prior to randomisation. We aimed, firstly, to assess first-time mothers' awareness of the recommended duration of exclusive breastfeeding and their intention to meet this recommendation, and, secondly, to explore the factors that are associated with the intention to exclusively breastfeed so that appropriate breastfeeding intervention strategies could be developed.

## Methods

### Study design

The design of the main study is a Randomised Controlled Trial (RCT), however for the purpose of this analysis we have used the data collected at the study baseline, which could be considered a cross-sectional survey. The RCT was conducted in southwest Sydney, Australia in 2008 and approved by the Ethics Review Committee of Sydney South West Area Health Service (RPAH Zone). The details of the HBT research protocol have been reported elsewhere [19].

### Study participants

All pregnant women who attended antenatal clinics of Liverpool and Campbelltown Hospitals located in south-western Sydney were approached by research nurses with a letter of invitation and information about the study. Women were eligible to participate if they were aged 16 years and over, were expecting their first child, were between weeks 24 and 34 of pregnancy, were able to communicate in English and lived in the local areas. Once eligibility was established and consent obtained, women then were asked to fill in a registration form with their contact information to allow the nurses to make further arrangements for the baseline data collection and random allocation to study group.

From around 2700 mothers who were approached, a total of 667 first-time mothers at 24-34 weeks of pregnancy were recruited for the main study. Four hundred and nine mothers were interviewed at their home before giving birth at the baseline and were included in this particular study. Another 258 mothers who also participated in the HBT were excluded, as we were not able to conduct the survey before they gave birth.

### Data collection and key measures

A face-to-face interview with participating mothers was conducted by one of four research nurses at their home, prior to randomisation. The interview lasted 20 to 30 minutes and included a range of questions in relation to the general health, physical activity and nutrition of the mothers, as well as demographic information.

To assess mothers' awareness of the breastfeeding recommendation and their intended duration of exclusive breastfeeding, they were asked the following questions:

"What do you understand to be the recommended age to which you should continue to exclusively breastfeed your child?"

"Do you plan to breastfeed your child?"

"To what age do you plan to exclusively breastfeed your child?"

In addition, the mothers were asked the main reasons for their decision to breastfeed, or not to breastfeed with an open-ended question. The face validity of the questions had been pilot-tested by some mothers and reviewed by breastfeeding experts in the field.

Other study variables, including age, employment status, education level, marital status, language spoken at home, and country of birth, were asked using the standard questions from the NSW Health Survey [20].

### Analysis

Statistical analyses were carried out using the computer package Stata 10 [21]. Relationships between study and outcome variables were examined using Pearson chi-square tests and Mantel-Haenszel chi-square tests for trend in proportions. Two logistic regression models were developed, one for awareness of breastfeeding recommendation and one for intention to meet the recommendation. Variables that were significant (P < 0.05) on bivariate analysis were entered into each model, then the least significant terms were progressively dropped until only those with P < 0.05 and those that confounded the effect of these variables remained in the model. Adjusted risk ratios (ARRs) with 95% confidence intervals were calculated by refitting the final models using log-binomial regression with the Stata binreg command.

## Results

The main characteristics of the participating mothers are shown in Table 1. The age range of the mothers was from 16 to 46 years with a mean age of 26 years. Most of the mothers (87%) were either married or living with their de facto partner. Twenty three percent had completed tertiary education and 11% spoke a language other than English at home. In addition, 21% were unemployed and 19% had paid maternity leave. Among those mothers who were in the workforce or studying, 11% planned to return to work or study after giving birth within three months, and a further 21% planned to do so within four to six months after giving birth.

**Table 1:** Characteristics of the 409 participating women and factors associated on bivariate analysis with awareness of the recommendation of exclusive breastfeeding for six months or the intention to meet the recommendations

**Characteristics**	**N = 409** **n^ (%)**	**Aware of recommendation of exclusive breastfeeding N = 249**		**Intend to meet the recommendation N = 170**	
			
		**n (row %)**	**P^#^**	**n (row %)**	**P^##^**
**Age**			< 0.001		0.24
<20	54 (13)	21 (39)		18 (33)	
20-24	121 (30)	71 (59)		48 (40)	
25-29	139 (34)	89 (64)		56 (40)	
30-34	62 (15)	42 (68)		33 (53)	
≥35	33 (8)	26 (79)		15 (46)	
**Marital status**			0.005		0.003
Married/de facto partner	354 (87)	225 (64)		157 (44)	
Never married	53 (13)	23 (43)		12 (23)	
**Employment status**			0.06		0.02
Employed	36 (9)	25 (69)		21(58)	
Paid maternity leave	77 (19)	57 (74)		38 (49)	
Unpaid maternity leave	111 (27)	70 (63)		50 (45)	
Unemployed	88 (21)	46 (52)		25 (28)	
Home duties	68 (17)	36 (53)		27 (40)	
Student	15 (4)	8 (53)		6 (40)	
Other	13 (3)	7 (54)		3 (23)	
**Education level**			< 0.001		0.25
Completed primary school to school certificate	86 (21)	38 (44)		28 (33)	
HSC to TAFE certificate or diploma*	229 (56)	138 (60)		98 (43)	
Completed University/tertiary education	92 (23)	71 (77)		43 (47)	
**Country of birth**			0.91		0.97
Australia	274 (67)	166 (61)		114 (42)	
Other	134 (33)	82 (61)		56 (42)	
**Language spoken at home**			0.68		0.24
English	364 (89)	220 (60)		148 (41)	
Other	44 (11)	28 (64)		22 (50)	
Anticipated time back to work or study after giving birth**			0.18		0.53
≤ 3 months	39 (11)	17 (44)		15 (39)	
4-6 months	75 (21)	44 (59)		25 (33)	
7-12 months	79 (22)	50 (63)		34 (43)	
After 12 months/don't plan to go back	141 (39)	89 (63)		64 (45)	
Don't know	26 (7)	18 (69)		11 (42)	
**Aware of recommended duration of exclusive breastfeeding**					< 0.001
Yes	249 (61)			153 (61)	
No	160 (39)			17 (11)	
**Intend to meet the recommendation**					
Yes	170 (42)				
No	239 (58)				

* HSC = Higher School Certificate, TAFE = Technical And Further Education

** Only for those who were in workforce or studying; ^ May not add up to 409 due to missing values

^# ^Comparisons of those who were aware and who were unaware of the recommendation

^## ^Comparisons of those who intended and who didn't intend to meet the recommendation

Among all 409 participating mothers, 61% knew the recommendation of exclusive breastfeeding for the first six months of life, and 39% either did not know or answered incorrectly. Only 42% of all mothers intended to meet the recommendation, however 94% (384) of the mothers did plan to initiate breastfeeding. Among the mothers who knew the recommendation, 61% intended to meet the recommendation, compared to only 11% among those mothers who were not aware of the recommendation.

Table 1 also shows factors associated on bivariate analysis with awareness of the recommendation of exclusive breastfeeding for six months or the intention to meet the recommendations. Awareness of the recommendation of exclusive breastfeeding for six months was significantly associated with older maternal age (Mantel-Haenszel χ^2^_1 _= 14.9, p < 0.001), marital status (married or de facto) (χ^2^_1 _= 7.9, p = 0.005), and a higher level of education (Mantel-Haenszel χ^2^_1 _= 20.3, p < 0.001). Marital status and employment status of the mothers were also found to be associated with their intention to meet the recommendation of exclusive breastfeeding for 6 months (χ^2^_1 _= 8.9, p = 0.003 and χ^2^_6 _= 14.8, p = 0.02 respectively). Awareness of the recommendation was very strongly associated with mothers' intention to breastfeed exclusively for six months (χ^2^_1 _= 103.6, p < 0.001).

The only factor associated with awareness of the recommendation on multivariate analysis was mother's level of education (Table 2). Mothers who had completed university/tertiary education were more likely to be aware of the breastfeeding recommendation than those who had school certificate or less, with an ARR after adjusting for the confounding effect of age of 1.45 (95% CI 1.08, 1.94, p = 0.02). More importantly, awareness of the recommendation was the only factor that was significantly associated with the intention to exclusively breastfeed for six months. Mothers who were aware of the recommendation were 5.6 times more likely to intend to breastfeed exclusively to six months (after adjusting for the confounding effect of employment status, ARR 5.61, 95% CI 3.53, 8.90, p < 0.001). Marital status was dropped in the final model as it was not significant after adjusting for education (p = 0.32), or after adjusting for awareness of the recommendation of exclusive breastfeeding (p = 0.27).

**Table 2:** Factors associated in multivariate analysis with awareness of recommendation of exclusive breastfeeding for six months or intention to meet the recommendation

**Variables**	**Awareness of the recommendation of exclusive breastfeeding**			**Intention to meet the recommendation**		
	
	ARR**	**95%CI**	**P**	ARR**	**95%CI**	**P**
**Age**			0.24^a^			
25-29	1					
<20	0.73	0.49 - 1.01				
20-24	0.96	0.79 - 1.18				
30-34	1.05	0.86 - 1.29				
≥35	1.16	0.93 - 1.44				
**Employment status**						
Unpaid maternity leave				1		0.19^c^
Employed				1.24	0.96 - 1.62	
Paid maternity leave				0.98	0.76 - 1.29	
Unemployed				0.72	0.50 - 1.01	
Home duties				1.06	0.80 - 1.40	
Student				0.99	0.58 - 1.71	
Other				0.61	0.25 - 1.51	
**Education**			0.02^b^			
Completed primary school to school certificate	1					
HSC to TAFE certificate*	1.20	0.91 - 1.58				
University or tertiary education	1.45	1.08 - 1.94				
**Aware of the recommendation of exclusive breastfeeding^e^**						< 0.001^d^
No				1		
Yes				5.61	3.53- 8.90	

* HSC = Higher School Certificate, TAFE = Technical And Further Education

**ARR = adjusted risk ratio

^a ^adjusted for education

^b ^adjusted for confounding effect of age

^c ^adjusted for awareness of the recommendation of exclusive breastfeeding

^d ^adjusted for confounding effect of employment status

The main reasons given by the mothers for planning to breastfeed or not breastfeed are summarised into several themes, showing some representative examples, in Table 3. The majority of the mothers understood that breastfeeding is good for the baby's and mother's health. For example:

**Table 3:** The main reasons given by the women for planning to breastfeed or not breastfeed

**Reasons for breastfeeding**	**Number (%) N = 384**	**Examples of what women said**
**Baby's health**	163 (42)	*Health, strength of immune system. Better for baby, everything right for baby.* *Healthy and nutritious for baby and less infection.* *Baby gets all nutrition. Baby's health, better than formula*. *Safest way to feed baby.* *More nutritious for baby, healthier. Formula can have chemicals and preservative.*

**Mother and baby's health**	38 (10)	*Better for baby, benefits for mother: weight loss, contraction of uterus.* *Good for mother and child. Prevent disease. Help prevent breast cancer in future.* *Antenatal class changed my mind. Back to normal weight quicker. Protector for breast cancer, can help prevent it. Boost immune system.*

**Cost effective**	52 (14)	*Good for baby. Cost effective.* *Best for baby, immunity, cheaper.* *Better for baby with immunity. Does not cost anything.*

**Convenient**	29 (8)	*Excellent for baby's health. Convenient.* *Immunity, no sterilizing, walking milk bar!* *Convenient, always there, no sterilization or preparation.* *Good for baby.*

**Bonding**	44 (11)	*Baby's health, bonding.* *Brings mother and baby closer, healthiest food for the baby.* *Good for baby, immunity, relationships with mother, growing, bonding.*

**Nature**	35 (9)	*Good for baby. God made milk special for a baby, natural, protect the baby from illness.* *That's what God planned for us to do: it's nature, all other animals do it. Best for baby.* *More nutritious. More benefits than formula. Mother generally prefers natural remedies.*

**All of them**	25 (6)	*Breastfeeding is best, nutrition, convenience, cheaper, bonding.* *Health benefits for baby. Convenience. Financial benefit.* *Natural.* *Good for baby, reduced risk of breast cancer, bonding, cheaper.* *Natural, cheaper, beneficial for baby, easier.*

**Reasons for not breastfeeding**	**Number (%) N = 25**	

**Back to work/no time**	7 (28)	*No time, have to work.* *Need to get back to work.* *No interest, going back to work soon.*

**Health concerns**	2 (8)	*Have an infection, unable to breastfeed. I would like to breastfeed.*

**Uncomfortable/embarrassment**	8 (32)	*I don't think I will feel comfortable. Too many people around.* *Did not feel comfortable.* *I would be embarrassed in public.*

**Convenient**	3 (12)	*It's easier to bottle feed. I don't know much about breastfeeding. My family all been bottle fed.* *More time consuming, hard enough already. It is easier to heat up a bottle.*

**Just don't want to breastfeed**	5 (20)	*Just did not want to breastfeed.* *Can't stand the thought of it. Freaks me out.*

"Breastfeeding is best, nutritious, convenient, cheaper and (helps the) bonding between mother and child."

"Breastfeeding can help mother back to her normal weight quicker, prevent breast cancer and boost the immune system."

"...Good for baby. God made milk special for a baby, (it's) natural, protects the baby from illness."

Mothers who did not plan to breastfeed (6%) gave reasons for not breastfeeding such as: *"No time, have to work"; "I would be embarrassed in public" *and *"Can't stand the thought of it. Freaks me out."*

Among the 25 mothers who did not plan to breastfeed, 21 were less than 24 years old, 10 were unemployed, only 15 completed the school certificate or less and only 9 knew the six months exclusive breastfeeding recommendation.

## Discussion

In this cross-sectional analysis we used data collected antenatally from 409 first-time mothers participating in the Healthy Beginnings Trial in southwestern Sydney. We found that a substantial proportion of the mothers (39%) did not know the current recommended duration of exclusive breastfeeding, and only 41% intended to meet the recommendation to breastfeed exclusively for six months. Awareness of the breastfeeding recommendation was significantly associated with the mother's intention to exclusively breastfeed her child. However, whether the intention to breastfeed is transferred to practice remains to be tested in future research.

Breastfeeding is widely acknowledged to have health benefits for mothers and babies [1-4]. Our study showed that most mothers were aware of some benefits of breastfeeding for both mothers and babies and, indeed, 94% of the mothers planned to breastfeed their child. In contrast, a small proportion of mothers who did not plan to breastfeed (6%) had strong negative attitudes towards breastfeeding. Changing negative feelings or negative perceptions of breastfeeding in this group of mothers is a challenge for breastfeeding promoters, but an important one, because they face greater health risks than the general community, being younger, less well educated and more likely to be unemployed.

To our knowledge, to date there is no research into mothers' awareness of the WHO breastfeeding recommendation and its association with the antenatal intention to breastfeed. Since the intention to breastfeed is a positive predictor for actual duration of breastfeeding [16-18], exploration of the factors influencing the intention may help health workers to address the issues related to breastfeeding intentions. A study by Forster et al revealed that breastfeeding intention was a strong indicator for breastfeeding initiation and duration across all groups of Australian women, including those with less formal education, younger women and those with less social support [16]. Therefore, focusing on mothers' intention to breastfeed may be an important strategy to increase breastfeeding rates and duration.

The negative effects of early weaning on children and mothers remain a significant public health concern. An analysis of data from the 2001 Australian National Health Survey found that fewer than 50% of infants were receiving breastfeeding milk at six months [22], which is considerably lower than the 80% figure recommended by the latest Australian Dietary Guideline for Children and Adolescents [3,22]. In addition, very few Australian infants are being exclusively breastfed for the recommended six months [8]. The lack of knowledge about the recommended duration of exclusive breastfeeding among first-time mothers in our study is likely to have contributed to a reduced number of mothers who intended to breastfeed exclusively for six months.

To date, most studies on breastfeeding awareness have focused on the health benefits of breastfeeding and infant feeding practices [4,7,9,10]. Few studies have looked into whether mothers actually understand the recommendations and what the recommendations mean to them. In interviews with some of the mothers participating in our study (data not presented), we found that they had limited understanding of the term "exclusive breastfeeding". In addition they expressed concerns about the quantity and quality of breast milk, and whether breast milk alone would be sufficient for their infant for six months. These findings were consistent with other studies suggesting that the most common reason cited by mothers for stopping breastfeeding was that the baby was unsettled, a behaviour often interpreted by mothers as indicating an insufficient milk supply [23]. This perception of insufficient supply appears to be due to a lack of information or lack of confidence regarding the normal process of lactation [24].

This study provides empirical evidence linking mothers' awareness of the breastfeeding recommendations and their intention to meet the recommendation. While our study had a relatively large sample of 409 first-time mothers, its generalisability is limited due to the locality of the study area. Southwest Sydney is the most socially and economically disadvantaged area of metropolitan Sydney [20]. We acknowledge the need to exercise caution in making assertions of the causal relationship between breastfeeding awareness and the intention to breastfeed based on a cross-sectional survey of this kind. Further studies are required to establish more definitively whether being aware of the breastfeeding recommendations actually improves intention to breastfeed and increases breastfeeding duration. In addition, whether model of pregnancy care plays a role in breastfeeding awareness and intention needs to be explored further.

## Conclusion

Potentially, our findings have a number of important policy implications for breastfeeding interventions. Efforts to encourage mothers to meet the recommendations should focus on improving mothers' knowledge and understanding of the recommendations, and address the concerns expressed by mothers about the quantity and quality of breast milk for the recommended duration. The effectiveness of targeted health promotion programs needs to be tested, particularly among young, unemployed and less educated mothers. There is a need to improve their perceptions, attitudes and knowledge, and also to change social norms in relation to breastfeeding practices.

In addition, appropriate public health policies to help mothers to breastfeed to at least six months, and to remove the barriers to breastfeeding, will be required to meet the WHO recommendations. The Australian Government's proposed 18 weeks paid maternity leave is a good start, but falls short of the required length, and falls particularly short of some Scandinavian and European countries' maternity leave entitlements of 50 to 64 weeks. For women who need to or choose to return earlier to work, the workplace needs to be able to provide child care and to facilitate breastfeeding on demand. Strategies recommended in the NSW Breastfeeding Policy Directive [25] provide examples for worksites on how to promote, protect and support breastfeeding in the community and amongst staff. These structural changes, along with health promotion programs which include changing social attitudes to breastfeeding, may improve the capacity of the most disadvantaged women to consider breastfeeding longer and more exclusively.

## Competing interests

The authors declare that they have no competing interests.

## Authors' contributions

LMW, LB, CR and GA conceived the HBT, and contributed to the development of the trial and the procurement of the funding.

In this study, LMW undertook literature review, data analysis and interpretation and wrote the original draft. JS provided advice on data analysis. LB, CR, GA and JS made significant comments on the draft. All authors have read and approved the final manuscript.
